# A High-Fat Diet Exacerbates the Course of Experimental *Trypanosoma cruzi* Infection That Can Be Mitigated by Treatment with Simvastatin

**DOI:** 10.1155/2020/1230461

**Published:** 2020-06-06

**Authors:** Débora Maria Soares de Souza, Guilherme de Paula Costa, Ana Luísa Junqueira Leite, Daniela Silva de Oliveira, Kelerson Mauro de Castro Pinto, Sílvia Elvira Barros Farias, Natália Figueiroa Simões, Nívia Carolina Nogueira de Paiva, Paula Melo de Abreu Vieira, Camilo Adalton Mariano da Silva, Vivian Paulino Figueiredo, Ana Paula de Jesus Menezes, Andre Talvani

**Affiliations:** ^1^Laboratory of Immunobiology of Inflammation, DECBI/ICEB, University of Ouro Preto, Brazil; ^2^Health and Nutrition Post-Graduate Program, ENUT, Federal University of Ouro Preto, Brazil; ^3^School of Physical Education, Federal University of Ouro Preto, Brazil; ^4^CBIOL/NUPEB, Federal University of Ouro Preto, Brazil; ^5^Tropical Medicine Post-Graduate Program, Federal University of Minas Gerais, Brazil

## Abstract

The protozoan *Trypanosoma cruzi* is responsible for triggering a damage immune response in the host cardiovascular system. This parasite has a high affinity for host lipoproteins and uses the low-density lipoprotein (LDL) receptor for its invasion. Assuming that the presence of LDL cholesterol in tissues could facilitate *T. cruzi* proliferation, dietary composition may affect the parasite-host relationship. Therefore, the aim of this study was to evaluate myocarditis in *T. cruzi*-infected C57BL/6 mice—acute phase—fed a high-fat diet and treated with simvastatin, a lipid-lowering medication. Animals (*n* = 10) were infected with 5 × 10^3^ cells of the VL-10 strain of *T. cruzi* and treated or untreated daily with 20 mg/kg simvastatin, starting 24 h after infection and fed with a normolipidic or high-fat diet. Also, uninfected mice, treated or not with simvastatin and fed with normolipidic or high-fat diet, were evaluated as control groups. Analyses to measure the production of chemokine (C-C motif) ligand 2 (CCL2), interferon- (IFN-) *γ*, interleukin- (IL-) 10, and tumor necrosis factor (TNF); total hepatic lipid dosage; cholesterol; and fractions, as well as histopathological analysis, were performed on day 30 using cardiac and fat tissues. Our results showed that the high-fat diet increased (i) parasite replication, (ii) fat accumulation in the liver, (iii) total cholesterol and LDL levels, and (iv) the host inflammatory state through the production of the cytokine TNF. However, simvastatin only reduced the production of CCL2 but not that of other inflammatory mediators or biochemical parameters. Together, our data suggest that the high-fat diet may have worsened the biochemical parameters of the uninfected and *T. cruzi*-infected animals, as well as favored the survival of circulating parasites.

## 1. Introduction

*Trypanosoma cruzi*, an intracellular protozoan parasite, is the causative agent of Chagas disease, which affects nearly 8 million people worldwide [1]. Multiple factors contribute to the clinical course of this infection, but the host immune response appears to be essential in coordinating the parasite replication and cardiac pathogenesis [2–4]. In this context, the production of cytokines and chemokines has been shown to essentially regulate leukocyte recruitment during *T. cruzi* infection, which could eliminate the parasite but also worsen the clinical condition [5–7].

Extrinsic factors such as elements present in the host diet or even medication might also contribute to the pathogenesis induced by *T. cruzi*. For instance, host lipid metabolism has been shown to have an important role in the host immune response and during infection by *T. cruzi*. This protozoan interacts with distinct components of the host metabolism to increase the lipid bodies in activated macrophages [8] and uses adipocytes as a chronic reservoir that can contribute to the up- or downregulation of inflammatory cytokines [9]. In addition, *T. cruzi* downregulates adiponectin secretion via peroxisome proliferator-activated receptor (PPAR) expression [10] and its *in vivo* invasion in animals fed a high-fat diet who present with high circulating levels of intracellular cholesterol is higher than it is in animals fed normal diets [11, 12]. Moreover, *in vitro*, the high-density lipoprotein (HDL) cholesterol of the host inhibits *T. cruzi* transsialidase activities by increasing parasitic infection and downregulating adiponectin release via PPAR expression [13].

Statins are an effective class of low-density lipoprotein (LDL) cholesterol-lowering agents, which act by inhibiting 3-hydroxy-3-methylglutaryl coenzyme A reductase [14]. Statins (e.g., simvastatin) also exert a cholesterol-independent immunomodulatory effect, which is likely mediated by preventing the production of isoprenoids, which act as critical lipid attachments for the posttranslational alterations of essential intracellular signaling proteins [15, 16]. We previously showed that mice and dogs treated with simvastatin exhibited reduced circulating inflammatory mediators and cell recruitment into *T. cruzi*-infected tissues [17, 18].

We assumed that some lipids and statins independently interfere with *T. cruzi* infection progression and the host immune response. Therefore, we performed experiments to prove our hypothesis that both extrinsic factors interact in C57BL/6 mice who were fed a high-fat diet (60% lipids), infected with the VL-10 of *T. cruzi*, and underwent simvastatin therapy (20 mg/day) for 30 days.

## 2. Material and Methods

### 2.1. Experimental Model, Parasite Infection, and Diets

Female C57BL/6 mice [19], 3-week-old age, were maintained at the Animal Facility of the Universidade Federal de Ouro Preto (UFOP) on a 12 h light/dark cycle with controlled temperature (22.0°C ± 2) and free access to water and food. Animals were fed a normolipidic diet (American Institute of Nutrition-93M) as the control diet and a modified high-fat diet containing 60% of calories obtained by lipids (PragSoluções Ltd., Sao Paulo-SP). The animals were fed for 2 months, and on day 60, they were infected with 5 × 10^3^ trypomastigotes of the VL-10 *T. cruzi* strain. The infection was confirmed by determining the daily parasitemia level by counting the parasites in 5 *μ*L blood samples obtained from the tail vein, according to the method of Brener (1962).

Before and after the infection, the animals were grouped (*n* = 10) as follows: uninfected, fed a normolipidic diet; uninfected, fed a high-fat diet; uninfected, fed a normolipidic diet and treated with simvastatin; and uninfected, fed a high-fat diet treated with simvastatin. The other infected groups were *T. cruzi*-infected and fed a normolipidic diet; *T. cruzi*-infected and fed a high-fat diet; *T. cruzi*-infected, fed a normolipidic diet, and treated with simvastatin, and *T. cruzi*-infected, fed a high-fat diet, and treated with simvastatin. See nutritional information on Table 1. Dietary intake was measured daily, and body mass gain was assessed weekly.

### 2.2. Treatment with Simvastatin

Simvastatin was administered at 20 mg/kg as previously standardized by our research group [18] and administered daily by gavage. Briefly, the treatment was started 24 h after infection and lasted for 30 days. The untreated animals received 0.05% carboxymethyl cellulose diluted in phosphate buffer solution by gavage while treated mice were administered simvastatin in the morning (7 : 30–8 : 00 a.m.) to coincide with the animals' cortisol peak.

### 2.3. Euthanasia and Tissue Collection

On day 90 after the infection, animals in their proestrus cycle were euthanized by an intraperitoneal overdose of ketamine (80 mg/kg) and xylazine (7 mg/kg) anesthetic and their blood and organs (the heart, liver, and abdominal fat tissue) were removed and one aliquot was stored in 10% formalin and another aliquot in a freezer at -80°C. The experimental protocol for this study was previously approved by the Ethical Committee for the use of Experimental Animals (CEUA) of the UFOP (Protocol #2012/42).

### 2.4. Total Cholesterol, HDL, LDL, and Triglycerides

The total cholesterol, HDL, and triglycerides were determined in plasma samples, using an enzymatic colorimetric kit for cholesterol, triglycerides, and HDL liquiform, from Labtest Diagnostica SA (Lagoa Santa, MG, BR). The LDL values were calculated using the following formula of Friedewald et al. [20], which is considered a reference by the Center for Disease, Control and Prevention (CDC): LDL cholesterol = total cholesterol − HDL cholesterol triglycerides/5.

### 2.5. Immunoassays

The circulating levels of CCL2, IFN-*γ*, IL-10, and TNF were quantified in the plasma and in homogenates of 50 mg of adipose or cardiac tissues, using an enzyme-linked immunosorbent assay (ELISA, PeproTech, Rocky Hill, NJ, USA). Briefly, 96-well microtiter plates were coated with the monoclonal capture antibodies at 100 *μ*L/well and incubated overnight at 25°C. After rinsing with the buffer (phosphate-buffered saline (PBS), 0.05% Tween-20), the nonspecific binding sites were blocked with 300 *μ*L/well of the blocking buffer (1% bovine serum albumin in PBS) for 1 h.

The samples were added (100 *μ*L/well), incubated for 2 h at room temperature, and then 100 *μ*L/well of the appropriate biotinylated detection antibodies diluted in blocking buffer containing 0.05% Tween-20 was added, followed by a 2 h incubation at room temperature. Avidin was added (100 *μ*L/well), and the plates were incubated for 30 min at room temperature. Finally, 100 *μ*L/well of the chromogen substrate 2,2′-alzino-bis (3-ethylbenzothiazoline-6-sulfonic acid (ABTS, Sigma-Aldrich Corp., St. Louis, MO, USA) was added, and the plates were incubated in the dark for 30 min at room temperature. Triplicate samples were read at 405 nm with wavelength correction set at 650 nm using a spectrophotometer (EMax Molecular Devices).

### 2.6. Total Hepatic Lipid Levels

The total hepatic lipids were extracted according to the method of Folch et al. [21]. In brief, 400 mg of the liver tissue was weighed into previously labeled plastic tubes, triturated with 8 mL of a chloroform-methanol (2 : 1, *v*/*v*) solution, and homogenized for 3 min at a speed of 10,000 rpm. The supernatants were transferred to new glass tubes of known weight, 2 mL 0.73% sodium chloride (NaCl) solution was added, the mixtures were homogenized and centrifuged again for 10 min at 3000 rpm, and the upper phase was discarded. The wall of each tube was washed three times with 1 mL Folch solution (3% chloroform solution, 48% methanol, 47% distilled water, and 2% NaCl), and the extracted lipids were oven-dried overnight at 60°C. After ensuring that the tubes were completely dry, they were weighed, and the amount of lipid extracted was calculated as the weight difference of the glass tubes before and after drying.

### 2.7. Histopathological Analysis

Fragments of the heart and inguinal adipose tissue were fixed in a methanol-dimethyl sulfoxide (DSMO) solution and embedded in Paraplast. Blocks containing these fragments were cut into 4 mm thick sections and stained with hematoxylin and eosin (H&E) for the quantification of inflammatory leukocytes. The stained sections were randomly evaluated at 40x magnification, in a total area of 74985 *μ*m^2^, the equivalent of 50 fields of analyzed myocardium and adipose tissue. Images were obtained using a Leica DM5000 B microchamber (Leica Application Suite, UK, version 2.4.0 R1) and processed using the Leica QWin (V3) image analyzer. For the myocardium, the inflammation was quantified by counting the number of cardiac cellular nuclei in the infected heart tissue compared with that in the uninfected mouse tissue.

### 2.8. Statistical Analysis

The data are expressed as the mean ± standard error of the mean (SEM) and were analyzed using the Kolmogorov-Smirnov normality test and Anova two-way followed by Tukey's posttest. All the analyses were performed using the PRISM 5.01 software (GraphPad, San Diego, CA, USA), and the level of significance was accepted at *p* < 0.05.

## 3. Results

In this present study, we monitored food intake and body mass gain and determined the total hepatic lipid levels of *T. cruzi*-infected animals receiving normolipidic and high-fat diets with or without simvastatin treatment, and the results are presented in Table 2. According to the data obtained, there was no difference in feed consumption and weight gain of the animals throughout the study. The animals fed with the normolipidic diet presented lower liver mass when compared to the animals treated with simvastatin. In this same group, the infection increased liver mass when compared to uninfected animals. In the group that received the normolipidic diet, there was also an increase in the liver mass in the association of infection with the treatment with simvastatin. When the animals were fed the high-fat diet, the infection also increased the body mass, and the same occurred in the infected animals treated with simvastatin. On the other hand, liver fat analysis showed an increase in fat deposition in this organ only among animals fed a high-fat diet, infected, and treated with simvastatin when compared to the same situation of the animals fed with the normolipidic diet.

**Figure 1:**
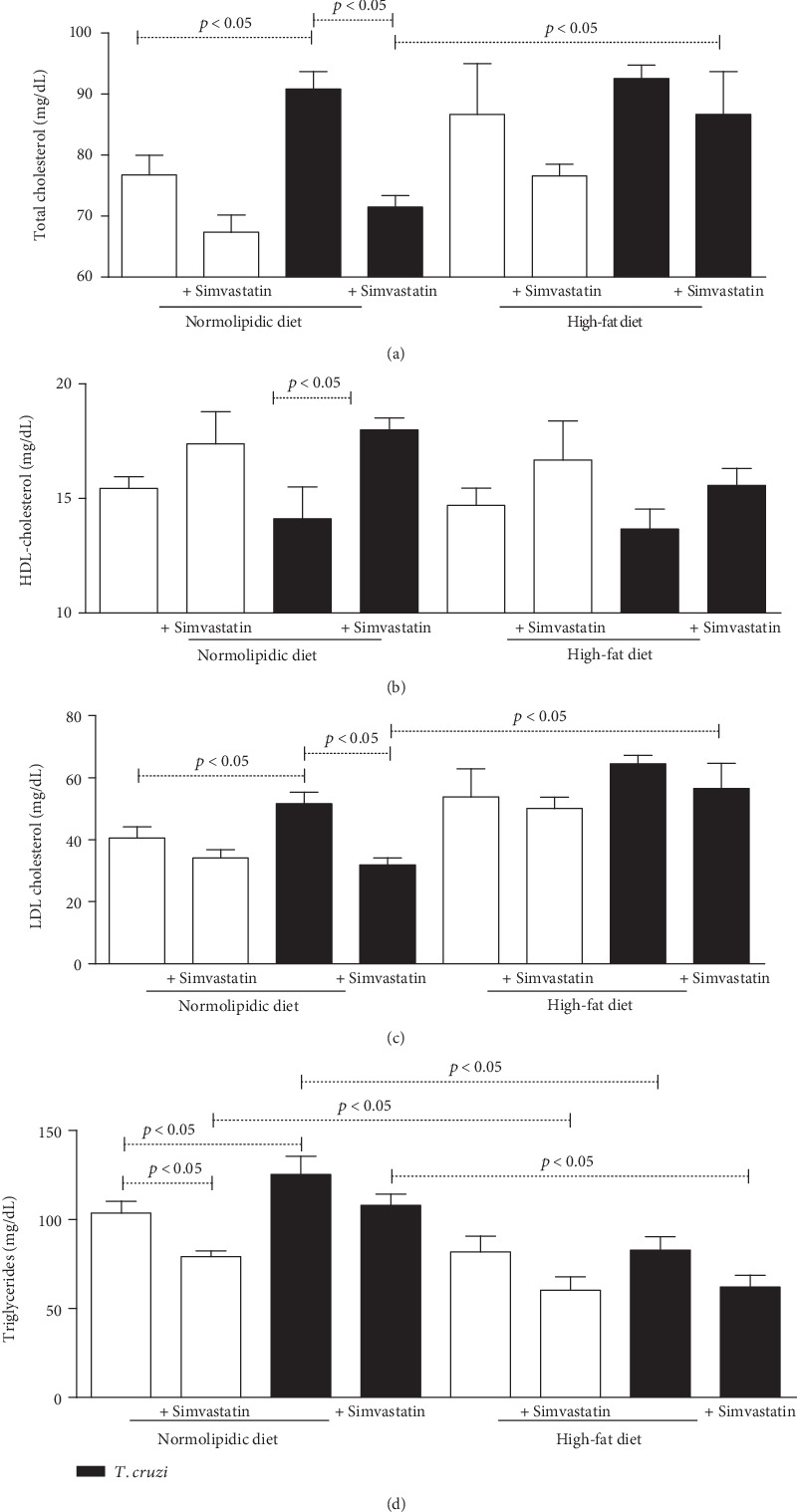
*Trypanosoma cruzi* increases total cholesterol, LDL, and triglycerides during acute phase of experimental infection. C5BL/6 mice were infected with VL-10 of *T. cruzi* and plasma used to measure total cholesterol (a), HDL cholesterol (b), LDL cholesterol (c), and triglycerides (d) using colorimetric enzymatic kits. Animals were fed with normolipid or high-fat diets and treated or not with simvastatin. White bars: uninfected animals; black bars: animals infected with *T. cruzi*. The data are mean ± standard error of the mean (SEM).

*T. cruzi* increased the total cholesterol (Figure 1(a)), the LDL cholesterol (Figure 1(c)), and triglycerides (Figure 1(d)) in mice fed with the normolipidic diet. In this context, simvastatin treatment was able to convert the pattern of total cholesterol and LDL cholesterol. In these *T. cruzi*-infected animals, the high-fat diet increased the total cholesterol and LDL cholesterol but neither HDL cholesterol (Figure 1(b)) nor triglycerides. Simvastatin failed in reducing the cholesterol and triglyceride values in the presence of *T. cruzi*.

**Figure 2:**
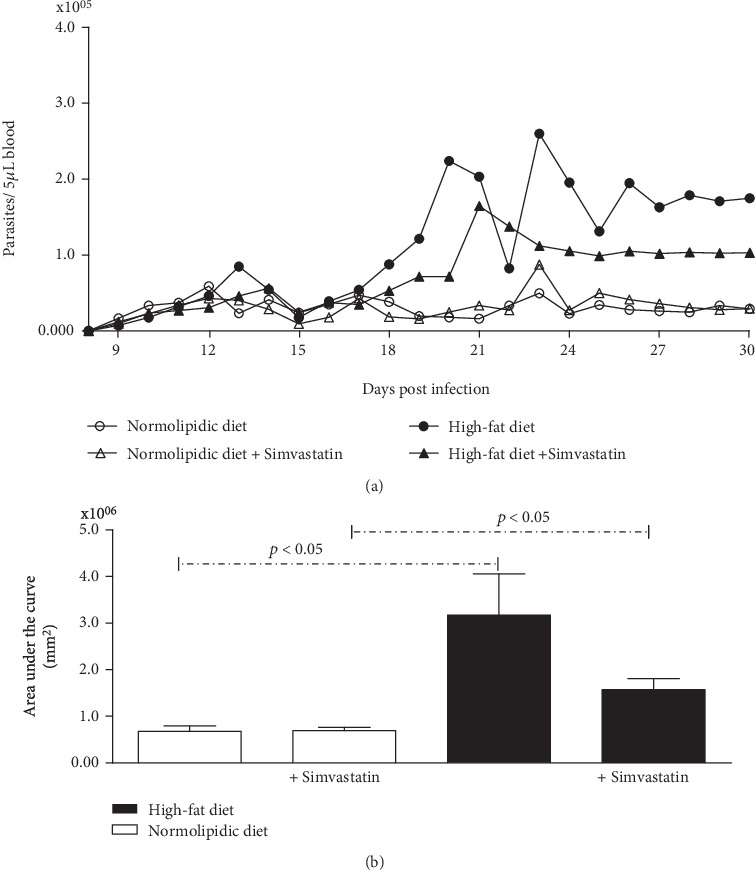
The high-fat diet increases the parasitemia curve. (a) Number of circulating parasites. White symbols: animals fed with normolipidic diet with and without simvastatin treatment; black symbols: animals fed with high-fat diet with and without simvastatin treatment. (b) Area under the parasitemia curve during 30 days of *Trypanosoma cruzi* infection in C57BL/6 mice. White bars: animals fed with normolipidic diet with and without simvastatin treatment; black bars: animals fed with high-fat diet with and without simvastatin treatment. Data are mean ± standard error of the mean (SEM).

The parasitemia curve (Figure 2(a)) and area under the curve analysis (Figure 2(b)) revealed that the high-fat diet contributed to a higher parasite amount in the blood of animals fed a normolipidic diet. In addition, simvastatin reduced the number of parasites in blood in the group fed a high-fat diet (50.40%), while in the group fed the normolipidic diet, no difference was observed.

**Figure 3:**
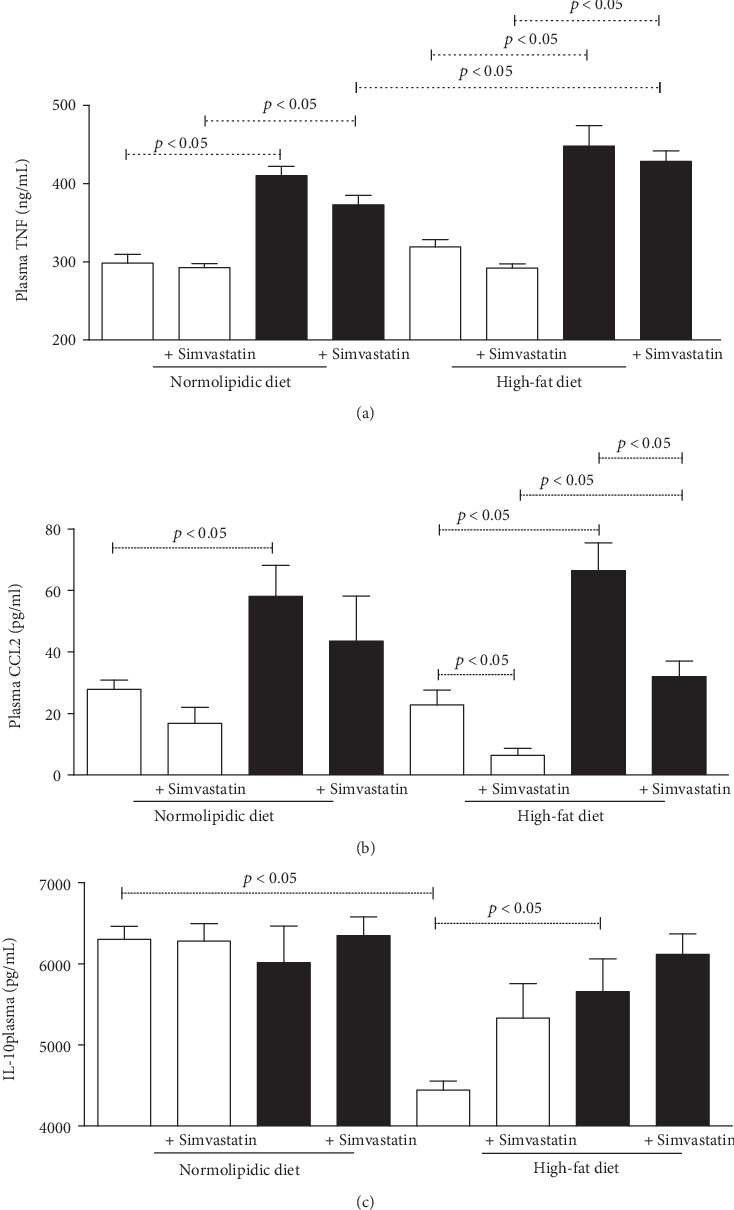
High-fat diet increases plasma TNF and CCL2 during the acute phase of experimental *T. cruzi* infection. TNF (a), CCL2 (b), and IL-10 (c) were measured by immunoassays in the plasma of C57BL/6 mice at 30 days of *T. cruzi* infection and after receiving normolipidic or high-fat diets. White bars: noninfected animals with and without simvastatin treatment; black bars: *T. cruzi-*infected mice with and without simvastatin treatment. Data are mean ± standard error of the mean (SEM).

We also observed an increase in the levels of plasma TNF in infected animals, and infected animals fed a high-fat diet had higher values of this cytokine when compared to infected animals fed a normolipidic diet (Figure 3(a)). Similarly, the infection elevated plasma CCL2 levels, and simvastatin worked by reducing this chemokine only in the group of infected animals fed a high-fat diet (Figure 3(b)). On the other hand, plasma IL-10 (Figure 3(c)) was higher in animals fed a normolipidic diet when compared to the high-fat diet, and treatment with simvastatin, as well as infection, increased levels of this interleukin in animals treated with a high-fat diet.

**Figure 4:**
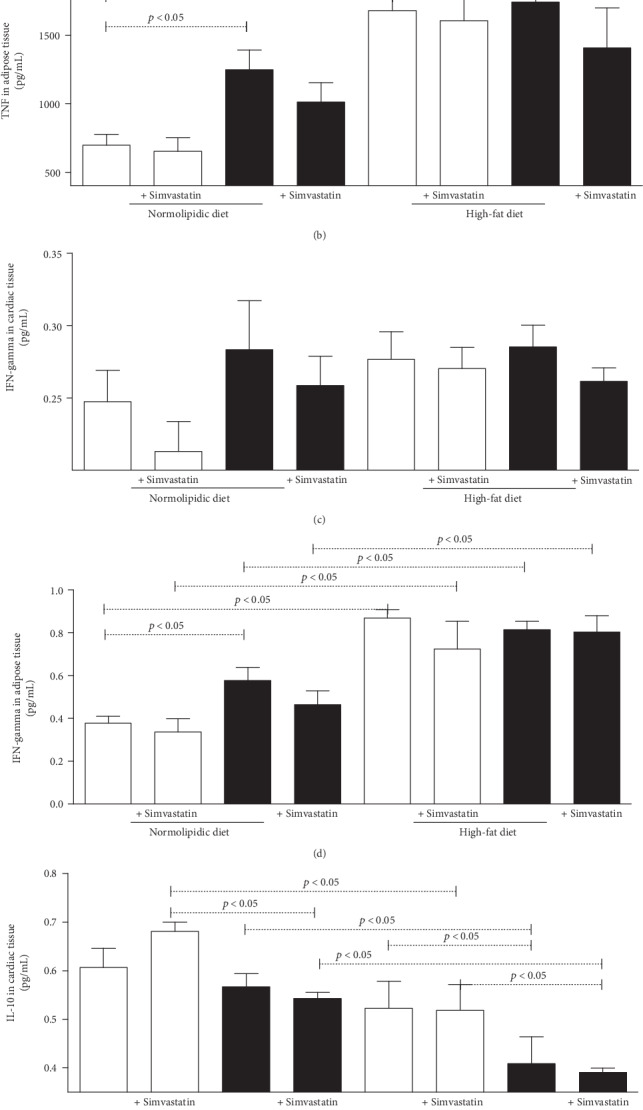
Biomarkers in cardiac or adipose tissues from C57BL/6 mice infected with the *Trypanosoma cruzi*. Uninfected (white bars) and *T. cruzi*-infected (black bars) mice were fed with normolipidic and high-fat diets and with and without simvastatin treatment. TNF (a, b), IFN-gamma (c, d), and IL-10 (e, f) were measured by immunoassay in 50 mg of cardiac or adipose tissues, respectively, after 30 days of infection. Data are mean ± standard error of the mean (SEM).

The cardiac tissue analysis showed higher TNF cytokine production among the infected and the high-fat diet mice when compared to the respective control group (Figure 4(a)). In the assessment of TNF in adipose tissue showed that regardless of infection, the high-fat diet stimulated high production of this cytokine (Figure 4(b)). On the other hand, there was no difference in the production of IFN-gamma in cardiac tissue (Figure 4(c)), but in adipose tissue, this cytokine had higher production in the groups fed with a high-fat diet when compared to the groups fed the normolipidic diet (Figure 4(d)). The production of IL-10 in cardiac (Figure 4(e)) and adipose (Figure 4(f)) tissues was higher in the groups fed the normolipidic diet, infected or not, when compared to the animals fed a high-fat diet. The animals fed the high-fat diet also had higher values of this cytokine when compared to the infected control.

**Figure 5:**
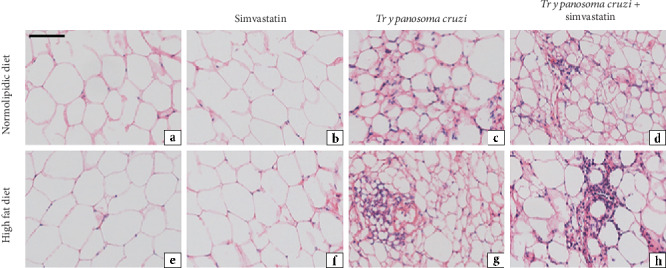
Representative photomicrographs of histological sections of adipose tissue from uninfected and infected C57BL/6 mice with *T. cruzi* (VL-10 strain). The acute inflammatory process was evaluated in the adipose tissue of mice infected with *T. cruzi* and fed with high-fat diet, under simvastatin treatment or not, as indicated in the figure. Tissues were stained with hematoxylin and eosin (H&E) at 30 days after infection. Scale bar = 50 mm.

Regardless of the diet (normolipidic control or high fat), the heart and adipose tissues of uninfected animals showed a normal histological appearance (Figure 5). Infected animals fed the normolipidic diet demonstrated a multifocal inflammatory process with moderate characteristics in the heart and discrete features in the adipose tissue that consisted mainly of mononuclear cells. Additionally, the infected animals fed the high-fat diet showed an increase in the inflammatory process compared with the infected animals fed the normolipidic diet that showed moderate to intense characteristics in the heart and discrete to moderate features in the adipose tissue.

**Figure 6:**
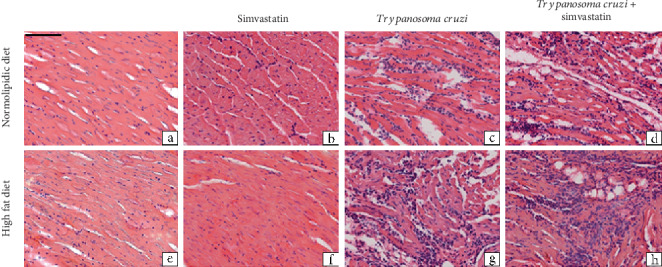
Representative photomicrographs of histological sections of heart tissue of mice infected with *Trypanosoma cruzi.* C57BL/6 mice were infected or not with the VL-10 strain of *T. cruzi* and fed, or not, with high-fat diet. In addition, some animals were treated with simvastatin and cardiac tissue extracted at 30 days after infection to processing and hematoxylin and eosin (H&E) staining. Scale bar = 50 mm.

In the heart (Figure 6), the atrial region showed higher concentrations of the inflammatory infiltrate while in the adipose tissue, areas with multilocular adipose cells were larger in animals fed the high-fat diet with or without the infection than they were in animals fed the normolipidic diet.

## 4. Discussion

Over the last few years, the role of adipose tissue has been highlighted in innate and adaptive immune responses as a modulator of the production of adipokines and other lipid mediators under infectious disease conditions [22, 23]. It is well-established that the inflammation caused by *T. cruzi* infection persists in distinct tissues and is highly dependent on the genetic background of the parasite and the immune response of the host, which determines the chronic pathogeny [24]. Thus, in *T. cruzi* infection research, adipose tissue and circulating fats have emerged as factors capable of controlling the adaptation of these protozoans and defining the course of the pathogenesis. In mammalian hosts, the adipose tissue also acts as a reservoir for protozoans in chronic stages of the infection. This phenomenon could be critical to the recrudescence of the infection under immunosuppressed conditions or may interfere with the development of cardiac and other associated metabolic disturbances [25, 26]. This present study provides key information that a high-fat diet affects the course of experimental *T. cruzi* infection by increasing the replication of the parasites; accumulation of fat in the liver, total cholesterol, and LDL levels; and circulating levels of inflammatory mediators. In addition and based on our previous experience where simvastatin exerted an immunomodulatory effect during the *T. cruzi* infection using the Y [17] and Colombiana [18] strains, this study also suggests a pharmacological action of simvastatin modulating the VL-10 strain infection of *T. cruzi*.

Generally, body lipids can be maintained at healthy levels by low-fat diet consumption, physical exercises, or pharmacological interventions such as statins, which also have an immunomodulatory property [27]. Therefore, an increase in the cholesterol levels and adipose tissue in association with the *T. cruzi* infection is thought to (i) benefit the parasites by providing the cholesterol and other lipids they require to maintain their metabolism, (ii) provide a dynamic cellular environment where parasites can hide, and (ii) elevate adipokines, which could somehow contribute to controlling the parasite by enhancing systemic and local inflammatory responses. We previously used simvastatin in experimental *T. cruzi* infection, but our focus was exclusively on the anti-inflammatory actions and potential amelioration of the clinical features. In these studied populations of *T. cruzi*, Y [17] and Colombiana [18] strains, they were able to induce high inflammatory responses in murine and dog models and, in these models, we demonstrated that simvastatin reduced the TNF, CCL2, and CCL5 levels, as well as the cardiac leukocyte infiltration and amastigote nests, which improved the echocardiographic parameters. In addition, a previous study showed simvastatin in association with benznidazole prevented cell adhesion molecule expression on endothelial cells during *T. cruzi* infection [28]. In this study, the simvastatin-induced 15-epi-lipoxin A4 prevented the ECAM expression on *T. cruzi*-infected EA.hy926 cells and blocked NF-*κ*B activation. It is worth stressing that *T. cruzi* presents high genetic variability which reflects characteristics concerning the natural history of infection, the biological behavior, the epidemiological patterns, and issues related to the diagnosis and treatment of this infection.

However, in our current study, we detected the simvastatin immunomodulatory effect on CCL2 plasma levels but not on other immunological parameters. Beyond lipid-lowering properties, simvastatin is known as an anti-inflammatory drug mainly through the inactivation of NF-*κ*B, a key activator of cytokine transcription and through the resulting decrease in the expression levels of reactive oxygen species, inhibition of matrix metalloproteinases, and decrease of proinflammatory cytokines including TNF. IFN-gamma and TNF are key markers released during the initial phase of *T. cruzi* infection to promote elimination and/or control of the parasite [5, 14, 15, 18]. The inflammatory response elicited by *T. cruzi* infection is accompanied by elevation of IL-10, which was not observed in the present study. A plausible explanation is based on the biological characteristic of the VL-10 strain. The VL-10 strain induces a moderate systemic and cardiac inflammation with a higher host survival than other strains, as previously demonstrated [29].

In this study, mice without simvastatin intervention fed a high-fat diet presented with alterations in parasitological and immunological parameters, which were beneficial to the circulating parasites. Specifically, this condition induced the accumulation of circulating total cholesterol and LDL and of fat in the liver. It has been demonstrated that a high-fat diet modified the immune response in uninfected mice by suppressing the cAMP response element-binding protein- (CREB-) PPAR-y signaling pathway, which could be associated with liver steatosis events [30]. In the presence or absence of infection, a high-fat diet was also shown to increase the detection of macrophages in fat tissue, production of reactive oxygen species, and mortality [31].

High-fat diet has been previously used in other studies of *T. cruzi* infection, but these involved different genetic variations of the parasite [9, 25]. It is worth noting that during the experimental *T. cruzi* infection, all variables of the different groups must be carefully considered to enhance the comprehension of the peculiarities and complexity of this infection. A hypothesis of the method by which parasites establish a close interaction with the adipose tissue is based on the metabolic characteristic of the adipocytes and their long lifespan (6–12 months) in the mammalian body. In the current study, we observed that the increase in LDL and total cholesterol levels related to the high-fat diet favored the survival of this protozoan. LDL has been shown to inhibit *T. cruzi* transsialidase in cultures of human fibroblasts in a concentration-dependent manner, which means that higher infection rate was observed with the addition of the LDL than without the LDL [32]. This observation could be a plausible explanation for the increased parasitemia in the experimental *T. cruzi* infection associated with the high-fat diet [33]. Furthermore, adipose tissue provides a suitable environment for replication of parasite [26].

Finally, unhealthy dietary habits have been strongly associated with body fat accumulation, cardiovascular disturbances, and untimely death in individuals from industrialized countries. The focus on the high-fat diets has now been extended to immune interventions, mainly associated with some tropical parasitic diseases such as *T. cruzi* infection. In summary, a high-fat diet could ameliorate the environmental for the *T. cruzi* parasite and worsen the biochemical and immunological responses in mammalian hosts. This fact can be illustrated with the present study, from the dosages of TNF, IFN, and IL-10 performed. In contrast, simvastatin is able to modulate the infection, since it acts on the low-density lipoprotein mechanisms, reducing the parasitemia and providing a protective heart effect. Therefore, further investigations would be necessary to enhance the understanding of how different populations of *T. cruzi* interact with elevated circulating fat levels in mammals and induce pathological alterations and how simvastatin contributes its protective effect.

## Figures and Tables

**Table 1:** Nutritional composition in 1 kilogram of diet.

Ingredients	Normolipidic diet (grams)	High-fat diet (grams)
Starch	619.4	249.4
Lard	-	320.0
Casein	14.0	190.0
Coline	2.5	2.5
Fiber	50.0	50.0
L-Cystine	3.0	3.0
Mineral mix	35.0	35.0
Vitamin mix	10.0	10.0
Soy oil	40.0	40.0
Sucrose	100.0	100.0
Total caloric value (*kilocalories*-kcal)	4,447.4	5,917.5

(I) Mineral mix (expressed in g/kg of the mixture): NaCl-139.3/KI-0.79/MgSO_4_·7H_2_O-57.3/CaCO_3_-381.4/MnSO_4_·H_2_O-4.01/FeSO_4_ ·7H_2_O–0.548/CuSO4·5H2O–0.477/CoCl_2_·6H_2_O–0.023/KH_2_PO_4_–389.0. (II) Vitamin mix (expressed in mg/kg of the mixture): retinol acetate–690.0/cholecalciferol-5,-/p-amino benzoic acid-10.0/inositol-10.0/niacin-4000.0/riboflavin-800.0/thiamine HCL-500.0; folic acid- 200.0/biotin-40.0/cyanocobalamin-3.0/dl-*α*-tocopherol-6.7/sucrose -q.s.p.1000.0. (III) BHT (Tert-Butylhydroquinone)-0.008 g to both diets/Kg ∗Added 1% of SIGMA® cholesterol conversion factors: proteins 4.0 kcal/g, lipids 9,0 kcal/g, sugars 4,0 kcal/g.

**Table 2:** The body parameters.

Parameters	Groups (mean ± std error)							
	Uninfected				*T. cruzi*			
	NLD	NLD+Simvas	HFD	HFD+Simvas	NLD	NLD+Simvas	HFD	HFD+Simvas
Food intake (g)	9.83 ± 0.18	9.65 ± 0.18	9.70 ± 0.53	9.66 ± 0.50	9.25 ± 0.39	9.46 ± 0.40	8.78 ± 0.27	9.10 ± 0.49
Body mass (g)	18.28 ± 0.72	17.63 ± 0.05	19.26 ± 0.56	19.83 ± 0.46	18.35 ± 0.51	19.09 ± 0.54	20.24 ± 0.57	20.17 ± 0.55
Liver relative mass (g)	0.041 ± 0.00*a*, *b*, *c*	0.045 ± 0.00*b*, *d*	0.048 ± 0.00*a*, *e*	0.045 ± 0.00*f*	0.058 ± 0.00*c*	0.060 ± 0.00*d*	0.061 ± 0.00*e*	0.061 ± 0.00*f*
Liver fat (g)	0.004 ± 0.0	0.002 ± 0.00	0.005 ± 0.002	0.004 ± 0.001	0.005 ± 0.001	0.003 ± 0.001*a*	0.008 ± 0.001	0.007 ± 0.001 *a*

Equal letters indicate difference between groups using Anova two-way and Tukey posttest (*p* < 0.05). NLD: normolipidic diet; HFD: high-fat diet. Simvas: Simvastatin.

## Data Availability

The data used to support the findings of this study are available from the corresponding author upon request.
